# Age-Dependent Effects of Yolkin on Contact Sensitivity and Immune Phenotypes in Juvenile Mice

**DOI:** 10.3390/molecules29143254

**Published:** 2024-07-10

**Authors:** Michał Zimecki, Jolanta Artym, Maja Kocięba, Ewa Zaczyńska, Angelika Sysak, Marianna Szczypka, Magdalena Lis, Aleksandra Pawlak, Bożena Obmińska-Mrukowicz, Katarzyna Kaleta-Kuratewicz, Aleksandra Zambrowicz, Łukasz Bobak

**Affiliations:** 1 Laboratory of Immunobiology, Department of Experimental Therapy, Hirszfeld Institute of Immunology and Experimental Therapy, Polish Academy of Sciences, R. Weigla Str. 12, 53-114 Wrocław, Poland; jolanta.artym@hirszfeld.pl (J.A.); maja.kocieba@hirszfeld.pl (M.K.); ewa.zaczynska@hirszfeld.pl (E.Z.); 2 Department of Pharmacology and Toxicology, Wrocław University of Environmental and Life Sciences, C. K. Norwida Str. 31, 50-375 Wrocław, Poland; angelika.sysak@upwr.edu.pl (A.S.); marianna.szczypka@upwr.edu.pl (M.S.); magdalena.lis@upwr.edu.pl (M.L.); aleksandra.pawlak@upwr.edu.pl (A.P.); bozena.obminska-mrukowicz@upwr.edu.pl (B.O.-M.); 3 Department of Biostructure and Animal Physiology, Wrocław University of Environmental and Life Sciences, C. K. Norwida Str. 25, 50-375 Wrocław, Poland; katarzyna.kaleta-kuratewicz@upwr.edu.pl; 4 Department of Functional Food Products Development, Wrocław University of Environmental and Life Sciences, J. Chełmońskiego 37 Str., 51-630 Wrocław, Poland; aleksandra.zambrowicz@upwr.edu.pl (A.Z.); lukasz.bobak@upwr.edu.pl (Ł.B.)

**Keywords:** juvenile mice, yolkin, contact sensitivity, ovalbumin, regulatory T cells

## Abstract

Yolkin, an egg yolk immunoregulatory protein, stimulates the humoral but inhibits the cellular immune response in adult mice. The aim of this investigation was to evaluate the effects of yolkin administration on the immune response using a model of juvenile, i.e., 28-day- and 37-day-old, mice. We examined the yolkin influence on the magnitude of the cellular immune response, which was determined as contact sensitivity (CS) to oxazolone (OXA), and the humoral immune response, which was determined as the antibody response to ovalbumin (OVA). Yolkin was administered in drinking water, followed by immunization with OXA or OVA. In parallel, the phenotypic changes in the lymphoid organs were determined following yolkin treatment and prior immunization. The results showed that yolkin had a stimulatory effect on CS in the mice treated with yolkin from the 37th day of life but not from the 28th day of life. In contrast, no regulatory effect of yolkin on antibody production was found in 28-day- and 37-day-old mice. Phenotypic studies revealed significant changes in the content of B cells and T cell subpopulations, including CD4+CD25+Foxp3 regulatory T cells. The association between the effects of yolkin on the magnitude of CS and phenotypic changes in main T- and B-cell compartments, as well the importance of changes in T-regulatory and CD8+ cells in the age categories, are discussed. We conclude that the immunoregulatory effects of yolkin on the generation of CS in mice are age dependent and change from stimulation in juvenile to suppression in adult mice.

## 1. Introduction

The maturation stage of the immune system in mammals determines their ability to generate humoral and cellular immune responses, which depend on the functional competence of T and B cells, as well as antigen-presenting cells. Neonatal mice already have the capability to mount an immune response, which is skewed to Th2 type [1]. The cells from the neonatal lymph nodes produce interferon gamma (IFN γ) and interleukin 4 (IL-4), but no IFN γ producing cells in the spleen are detected, and Th-1 memory effector function is impaired [2]. In addition, the Th1-mediated, but not the humoral immune response, is suppressed in weanling mice administered a poor diet [3,4], which represented additional proof for the dominance of a Th2 type immune response in young mice. The antigen-presenting function of splenic B cells to T cell lines is absent in neonatal 6–8-day-old mice [5] and gradually increases by six weeks of age [6]. With regard to peritoneal B1 cells, which are responsible for initiation of contact sensitivity [7,8], the neonatal 5–6-day-old mice contain mainly B1 cell progenitors [9], which attain a fully mature phenotype at 10 weeks of age. In addition, the ability of antigen-presenting Langerhans cells in the skin to carry antigens to lymph nodes is not fully efficient until 14 days of age [10]. Nevertheless, the full antigen-presenting ability of B cells and dendritic cells to initiate an antibody response to a bacterial vaccine is already achieved at the 4th week of age [11]. Delayed-type hypersensitivity responses are mediated by antigen-specific CD4+ and CD8+ T cells [12,13] and controlled by suppressor CD8+ T cells [14] and CD4+ CD25+ regulatory T cells [15,16,17].

Maturation of the immune system function may be elicited by a variety of peptides and proteins, including thymic factors [18,19], lactoferrin [5,20], colostrum peptides [21,22], or plant-derived compounds [23]. Recently, a protein isolated from egg yolk attracted much interest [24]. Yolkin is a degradation product of vitellogenin, an evolutionary old protein precursor, present in all egg-laying animals and is essential for the development and protection of embryos [25]. The biologically active fraction consists of a glycosylated protein with a molecular mass of 35 kDa and a set of smaller peptides. Yolkin is a potent inducer of both pro- and anti-inflammatory cytokines [24,26,27,28]. On the other hand, the protein exhibits anti-inflammatory properties in lipopolysaccharide (LPS)-induced nitric oxide production, cellular lipid peroxidation, and cytokine production [26,27]. Indirect evidence has been provided for yolkin mediating its immunoregulatory actions via the Toll-Like Receptor 4 (TLR4) receptor [26]. It is highly possible that the actions of yolkin administered via the oral route are initiated when the protein interacts with TLR4 present in epithelial cells in the oral cavity [29] and gut [30]. Therefore, to determine phenotypic changes during oral yolkin administration, mesenteric lymph nodes belonging to the gut-associated lymphoid tissue (GALT) were used in our study.

Yolkin also shows antiviral activity [26] and improves pro-cognitive functions in rats [31], probably in association with its ability to induce production of brain-derived neurotropic factor [28]. The effects of yolkin on the immune response in mice was also investigated [32]. Yolkin stimulated the humoral immune response to sheep red blood cells (SRBC) but inhibited the cellular immune response determined in a contact sensitivity (CS) to oxazolone (OXA) test. The ex vivo and in vitro experiments also showed that yolkin promoted differentiation of immature T and B cell lines as well as T and B cells in the lymphoid organs [32]. Of interest, yolkin injected in ovo into developing embryos on the 18th day of egg incubation increased the content of T cells in the spleen and the circulating blood, as well as IL-1β and IL-2 levels in the circulation of adolescent hens [33].

In summary, the accumulated data regarding the biological properties of yolkin clearly indicate its ability to promote the maturation of immune system cells. Despite reasonably well-recognized changes in the phenotype and function of immune cells in neonatal and weanling mice, virtually no information exists regarding the cellular immune response in the age interval from weanling to early adolescent mice.

According to our extensive experience, we have not been able to generate a satisfactory high delayed-type hypersensitivity response to several antigens in various strains of mice younger than 8 weeks. Therefore, we assumed that yolkin could accelerate the development of a cellular immune response in adolescent mice. The aim of this investigation was to evaluate the effects of yolkin administered in drinking water to two groups of juvenile mice from the 28th day of life and from the 37th day of life (referred to later as 28- and 37-day-old mice), on several parameters characterizing CS to OXA and antibody production to ovalbumin (OVA). In the discussion, we also took advantage of our unpublished results regarding the effects of yolkin on CS in adult 8–10-week-old BALB/c mice from other experimental projects, bearing in mind that the effect of the intraperitoneal (i.p.) administration of yolkin in this model was inhibitory [32]. The experiments also involved the determination of phenotype changes in major immune cell types following yolkin administration, prior to immunization, in an attempt to correlate yolkin-induced phenotypic changes with its effect on the magnitude of CS to OXA and antibody production to OVA.

## 2. Results

### 2.1. Effects of Yolkin on Contact Sensitivity to Oxazolone in Adolescent Mice

**Figure 1:**
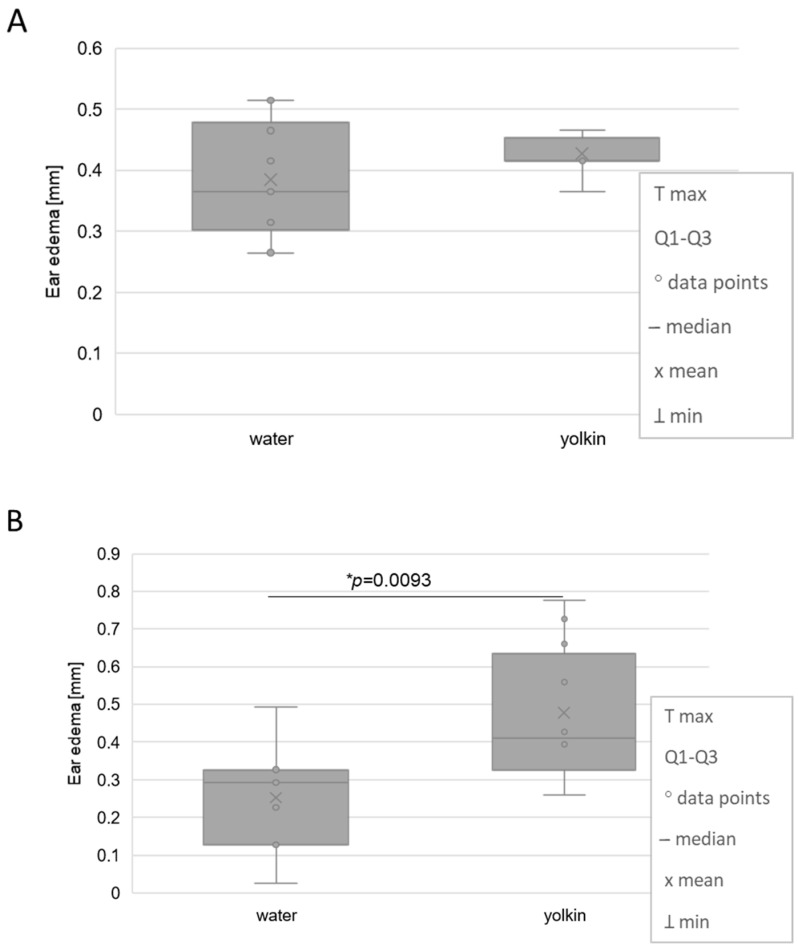
The effects of oral administration of yolkin to 28-day-old (**A**) and 37-day-old (**B**) juvenile mice on their contact sensitivity to oxazolone measured by the antigen-specific ear edema. Mice with a mean age of 28 days or 37 days were administered yolkin in drinking tap water, at a concentration of 5 µg/mL, for 15 consecutive days. The next day (on 16th day of the test), mice were sensitized with 0.5% OXA on their shaved abdomens. The CS reaction was elicited 5 days later by application of 1% OXA on the ears. The ear edema was measured 24 h later using spring caliper. An antigen-specific increase in ear thickness (BG is subtracted from the responses measured in sensitized mice) is presented as a mean and median values of ear thickness measured in 5 mice (10 determinations) in mm. * statistically significant in comparison to control, water-drinking mice.

In our previous study on the effects of yolkin on the cellular and humoral immune response, we found that yolkin, administered i.p. in four doses, before sensitization with OXA to 8–10-week-old mice, at doses of 1 mg/b.w. or 0.1 mg/b.w., inhibited the ear edema by 32% and 48% (a statistically significant effect), respectively [32]. Here, we intended to evaluate the effect of yolkin administration in drinking water on the development of CS in adolescent mice of an average age of 28 and 37 days. The results show that the magnitude of CS, determined by the auricle swollenness, was stimulated (by 51%) in mice which were treated with yolkin from the 37th day of life but very weakly in 28-day-old mice (Figure 1). In addition, the measurements of the ear edema derived from control groups of other experimental projects in adult mice treated with yolkin at a later age (8-weeks old) revealed that yolkin barely inhibited CS, i.e., by 2% at i.p. and by 6% at per os treatment.

Other investigated parameters in these groups of mice, such as the weight of thymuses, spleens, and drained lymph nodes, as well as their cellular content, underwent some, but not statistically significant, variations.

### 2.2. Effects of Yolkin on Blood Cell Type Composition in Contact Sensitivity to Oxazolone in Adolescent Mice

Table 1 shows, additionally, that on the day of measurement of the ear edema, yolkin-treated 37-day-old mice had higher levels of blood circulating neutrophils (by 5.5%) but a lower percentage of eosinophils (1% vs. 2%) and monocytes (1.9% vs. 3.8%). The observed changes between the control (water-treated) and yolkin-treated groups were not statistically significant.

### 2.3. Effects of Yolkin on the Phenotype of Cells in the Lymphoid Organs

The data presented in Table 2 revealed no statistically significant changes in the T cell subpopulations in the spleen of juvenile mice treated with yolkin from the 28th day of life, although a 6.65% increase in B cell content in the spleen was registered. Also, no change in splenic macrophage as well as in CD19+ cell level in the bone marrow was found. In contrast, significant changes in T cell subpopulations and B cell content were found in mesenteric lymph nodes and splenic regulatory T cells. In the lymph nodes, a significant increase in the percentage of all T cells (CD3+), CD4+, and CD8+ subpopulations were registered, with a concomitant decrease in CD19+ B cells. In turn, the content of splenic regulatory CD25+Foxp3+ T cells was lowered by about 20% in comparison to the control group.

The phenotypic changes in juvenile mice treated from the 37th day of age with yolkin were different from those observed in mice treated with yolkin 9 days earlier (Table 3). First, changes in the mesenteric lymph nodes only occurred in restricted CD8+ (a significant decrease). The phenotypic alterations found in the spleen regarded only a significant drop in the percentage of T-regulatory cells. In both age categories no changes in the content of cells bearing the macrophage or dendritic cell phenotype were found.

### 2.4. Effects of Yolkin on the Antibody Production to Ovalbumin

**Figure 2:**
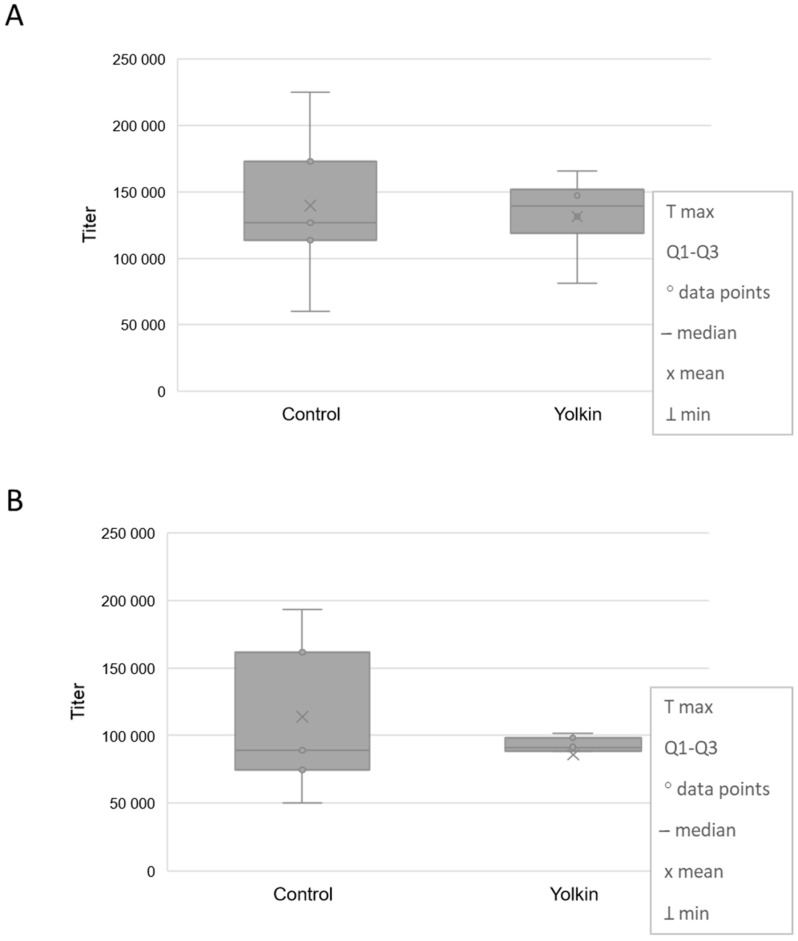
The effects of oral administration of yolkin to juvenile 28-day-old (**A**) and 37-day-old (**B**) mice on the humoral immune response measured by antibody production to ovalbumin. Mice with a mean age of 28 days or 37 days were administered yolkin in their drinking tap water, at a concentration of 5 µg/mL, for 15 consecutive days. The next day (on 16th day of the test) mice were sensitized s.c. with OVA suspended in iFa and, after 4 days, received s.c. a booster dose of OVA. On day 21 after immunization, the mice were bled and the IgG1 antibody level was determined in serum. Mean values of antibody titer for each group are shown.

The results presented in Figure 2 indicate that treating mice with yolkin did not change the IgG1 antibody titer to OVA in both age categories. Similarly, the whole IgG antibody titer was not changed by yolkin.

## 3. Discussion

In this work, we showed that the effectual phase of CS to OXA in adolescent mice, treated orally with yolkin, was stimulated in 37-day-old mice, with only a tendency for stimulation of CS in 28-day-old mice. However, the treatment of both juvenile groups of mice with yolkin had no effect on the antibody titer to OVA. Moreover, the treatment of mice with yolkin led to phenotypic changes in immune cells in the lymphoid organs, which differed in these two age categories.

The involvement of mature T cells bearing the CD4+ and CD8+ phenotype in the generation and control of delayed hypersensitivity is complex, since both T cell phenotypes may characterize T cell subsets responsible for initiation and control of this type of the immune response. The phenotypic determination of T cells presented in this work, except regulatory T cells, does not differentiate between innate CD8+ suppressor cells in lymphoid organs [34,35] and effector CD8+ cells [12,14,36] present at the site of antigen-specific inflammation. The number of effector CD8+ cells cooperating with antigen-specific CD4+ [13] and present at the site of inflammation is probably very small and escapes our determination in this work, unless stained by an immunohistochemical method. Thus, the interpretation of the association between phenotypic changes and the magnitude of the effectual phase of CS is difficult. However, the inclusion of T-regulatory CD25+Foxp3+ cell determination [15,16,17] facilitated an interpretation of the association between the phenotype and the magnitude of the CS reaction. Innate CD8+ T cells and regulatory T CD25+Foxp3+ cells may play similar roles in the control of CS. In both juvenile mouse categories, the lymph node levels of T-regulatory cells were downregulated by yolkin, but CD8+ cell content was elevated in the mice treated with yolkin from the 28th day of life and was lowered in the group treated from the 37th day of life. Thus, an interplay between the actions of these T cell subsets could account for the differences in the effects of yolkin on CS. Although the lowered T-regulatory cell content could upregulate the CS response, the increase in the innate CD8+ cell level probably weakened the action of T-regulatory cells, as observed in the mice drinking yolkin from the 28th day of life. Apart from the regulatory role of CD8+ and T-reg cells in mediating yolkin’s effects on CS in juvenile mice, the status of antigen-presenting cells and other accessory cells in generating CS in this age category may also be important. The failure of yolkin to significantly stimulate CS in 28-day-old mice may result from B1 cells not being fully competent, playing a crucial role in the initiation of CS in this mouse category [9]. Nevertheless, a very recent study in a homologous model showed that yolkin, administered in ovo to 18-day-old embryos, strongly elevated CD4+ T cell content and not B cells in adolescent 35- and 42-day-old chickens [33]. This supports our findings that at an early stage of ontogenic development yolkin promotes the development of the T cell compartment.

The interpretation of the results in the mice that drank yolkin from the 37th day of life may be more straightforward, since the content of both the suppressive types of T cells (CD8+ and CD25+Foxp3+) were lowered, which could result in stimulating the CS response in this age category. The determination of blood cell composition in the mice treated with yolkin from the 37th day of life correlated with a registered increase in the ear edema because the content of neutrophils, responsible for infiltration of inflamed dermis, was elevated. These changes also indicate an increase in the overall state of yolkin-treated mice. In addition, there was a decrease in the percentage of eosinophils—cells associated with Th-2 type response.

In the interpretation of our results on CS to OXA in the juvenile mice, a possible involvement of macrophage subsets of different secreted cytokine profiles may also be helpful. It appears that yolkin preferentially promoted the development of M1 macrophages [37] and that the macrophage phenotypes in juvenile mice are mixed, although strongly skewed towards the M1 type [38]. This may explain the potential of yolkin to regulate proportions of these macrophage subsets, which, in the case of mice treated with yolkin from the 37th day of life, could promote M1 expansion, producing Th1-type cytokines and, consequently, delayed-type hypersensitivity to OXA. Nonetheless, no differences in the splenic macrophage contents in both age categories were found.

In adult mice of a more advanced age (2.5-months-old) [32], the regulation of CS to OXA by yolkin was dose-dependent, with a strong suppressive effect at a dose of 0.1 mg/b.w. A possible explanation for the suppressive effect of yolkin in adult mice is that in the process of aging, TLR4 agonists, such as LPS, redirect cytokine production from the pro- to anti-inflammatory IL-10 [39], which inhibits CS. Such a scenario may occur in the case of yolkin, a TLR4 agonist. Another characteristic feature associated with the effects of yolkin in adult mice was a change in the proportion between B CD19+ cells and all T cells in the spleen and mesenteric lymph nodes [32]. A strong decrease in the content of the T cell compartment, registered in this study, which participates in the generation of CS, may, in part, explain the suppressive effect of yolkin on CS in adult mice.

Our previously conducted experiments in other experimental models in adult 8-week-old mice, also involving the CS to OXA model and yolkin, revealed that the effects of yolkin on ear edema and histologically determined auricle or epidermis thickness were virtually non-existent or slightly inhibitory, irrespective of the route of yolkin administration (i.p. or per os). In addition, no effect of yolkin on the percentage of T-regulatory cells was found.

In summary, we may cautiously conclude that yolkin stimulates CS in a narrow time window in juvenile mice (37-day-old mice), followed by a lack of significant regulation in adult 8-week-old mice, and suppression at a more advanced age (10-week-old mice) [32]. In this age category, yolkin did not affect T and B cell levels and its stimulatory effect on CS was correlated with the decreases in T-regulatory and CD8+ T cells. In the youngest mice (28-day-old), the non-significant stimulatory action of yolkin may be, in part, caused by the insufficient competence of the antigen-presenting cells. Additionally, the resulting regulatory effect of yolkin on CS in these mice may be due to the yolkin-elicited changes in the proportions of innate and T-regulatory cells.

The in vivo effect of yolkin on the recruitment of B cells in the bone marrow was modest, albeit with a stimulatory tendency. However, in in vitro experiments, yolkin had significant effects on the enlargement of the B cell pool in bone marrow cells in 3-to-4-week-old mice, indicating that it may directly affect the bone marrow milieu in weanling mice. Considering the advanced maturation stage of the B cell compartment in newborn and weanling mice, the lack of significant effects on B cell content after yolkin treatment in the juvenile may not be surprising. Instead, the percentage of T cells in the lymph nodes of 28-day-old treated mice significantly increased, although with no apparent effects on the magnitude of CS.

The lack of stimulation of the humoral immune response in juvenile mice could occur for two reasons. First, as described in the introduction, the immune system of mice at the end of treatment with yolkin was fully competent in developing antibody production, and second, the immunization protocol was designed to induce maximal antibody levels, so the regulatory nature of yolkin could not be revealed. Perhaps the differences between the mice categories could be found at suboptimal immunization protocols.

We are aware of the limitations regarding final, firm conclusions that can be drawn from the obtained data. Ideally, this study should be performed in parallel in several age categories, including weanling, juvenile, young, and adult mice. However, such a full-scale study would be very difficult for many reasons. Nevertheless, we took advantage of the already published and previously obtained data in an attempt to describe changes in the effects of yolkin on the development of cellular and humoral immunity in mouse ontogeny, in association with phenotypic alterations.

In summary, we first conclude that yolkin differentially promotes B and T cell compartments depending on age. Second, yolkin stimulates the T cell-mediated cellular immune response in the CS model in adolescent mice, whereas in adult mice its effect is suppressive. In contrast, no stimulatory effect of yolkin is observed in juvenile mice with regard to generating antibody production. This is a preliminary report pertaining to the age-dependent effects of yolkin on the immune status of mice, which should be verified in other experimental models.

## 4. Materials and Methods

### 4.1. Mice

Three-week-old BALB/c female mice were purchased from Envigo, Netherlands, and from the Animal Facility of Mossakowski Medical Research Centre, Polish Academy of Sciences, Warszawa, Poland. The mice were housed in cages at 21–22 °C with a 12/12-h light/dark cycle and were fed a commercial, pellet food as well as water ad libitum. The Local Ethics Committee at the Institute of Immunology and Experimental Therapy, Polish Academy of Sciences, Wrocław, Poland, approved this study (permission # 003/2022).

### 4.2. Reagents

Hanks’ balanced salt solution (HBSS), phosphate-buffered saline (PBS), bovine serum albumin (BSA), 2 M H_2_SO_4_, May–Grünwald and Giemsa stains, Histopaque 1077 g/mL, incomplete Freund’s adjuvant (iFa), oxazolone (OXA), and ovalbumin (OVA) were from Merck (Munich, Germany); rat anti-mouse CD19+FITC/CD3+:RPE (Bio-Rad Laboratories, Hercules, CA, USA, DC035), rat anti-mouse CD4+:FITC/CD8+:RPE (Bio-Rad, DC034), rat anti-mouse CD14:RPE (Invitrogen, Waltham, MA, USA, AB-465563), and rat anti-mouse CD169:FITC were from (Bio-Rad, MCA884F); hamster anti-mouse CD11c:Alexa488 was from (Bio-Rad, MCA1369A488); mouse regulatory T cell staining kit #1 was sourced from (eBioscience, San Diego, CA, USA).

### 4.3. Yolkin Preparation

Yolkin was isolated from hen egg yolks according to an originally described procedure [24]. A detailed procedure of isolation and SDS-PAGE analysis of the yolkin preparation was recently described [32]. The yolkin preparation consisted of three main proteins with MWs lower than 45 kDa.

### 4.4. Experimental Design

Mice with a mean age of 28 days or 37 days were administered yolkin in their drinking tap water, at a concentration of 5 µg/mL, for 15 consecutive days. The concentration of yolkin was calculated based on effective oral dose in a rat study [31], estimated as approximately 30 µg/rat/day. In our model, the yolkin concentration corresponded to 25 µg/mouse/day, taking into account the estimated daily intake of water of 5 mL/mouse. The duration of the treatment was based on the assumption that the mice should be treated with yolkin until they reach an age of immune competence (i.e., of 6–7 weeks).

**Scheme 1:**
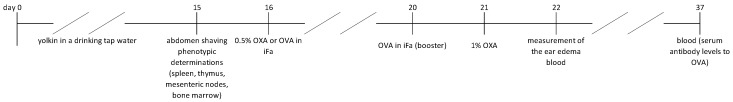
The experimental design.

The yolkin solution was replaced every 24 h. The control mice drank only fresh, filtered tap water. On day 16 of the test, some of the mice were sacrificed for determination of cell phenotypes in the lymphoid organs. Other groups of mice were sensitized with OXA for determination of contact sensitivity (CS) on day 5 (cellular immunity) or sensitized with OVA for measurement of serum antibody levels to OVA following 21 days (humoral immunity). The experimental design is presented in Scheme 1.

### 4.5. Phenotypic Determinations of Splenocytes, Mesenteric Lymph Node Lymphocytes, and Bone Marrow Cells

The mice were anesthetized with isoflurane and sacrificed by cervical dislocation. The lymphatic organs (spleens, femurs, and mesenteric lymph nodes) were isolated and passed through a nylon mesh into 1 mL of sterile, ice-cold PBS. Bone marrow cells were flushed out from the femurs using a syringe with 4 mL of ice-cold HBSS. Cell suspension was passed through the nylon mesh to remove debris. The number of mononucleated cells from central and peripheral lymphatic organs was counted in a Thoma hemocytometer using a Türk solution. Splenocytes and mesenteric lymph node cells were centrifuged (2250× *g*, 15 min, 4 °C) on a layer of Histopaque 1077 g/mL. After centrifugation, the cells were collected from the interphase and washed twice (375× *g*, 8 min, 4 °C) with sterile, ice-cold PBS supplemented with 1% BSA. The cells from the bone marrow were washed twice (375× *g*, 8 min, 4 °C) with a sterile, ice-cold HBSS. After the second wash, the cells were suspended in PBS with 1% BSA at a density of 1 × 10^7^ cells/mL. The viability of each cell suspension was 90–98% according to a trypan blue dye-exclusion assay. The cells were re-suspended in 100 μL PBS containing 1% BSA.

The lymphocytes from the spleen and mesenteric lymph nodes (1 × 10^7^ cells/mL) were stained with rat anti-mouse CD19+:FITC/CD3+:RPE dual-color reagent, and rat anti-mouse CD4+:FITC/CD8+:RPE dual-color reagent at the dilutions recommended by the manufacturer. The splenocytes were also stained with rat anti-mouse CD14:RPE and rat anti-mouse CD169:FITC monoclonal antibodies at the dilutions recommended by the producer. Bone marrow cells were stained with rat anti-mouse CD19+:FITC/CD3+:RPE dual-color reagent and hamster anti-mouse CD11c:Alexa488 at the dilutions recommended by the producer. After incubation and the washes described above, fluorescence was estimated using a flow cytometer (FACS Calibur; Becton Dickinson Biosciences, San Jose, CA, USA), and lymphocyte marker distribution was analyzed using CellQuest Pro, version 6.0. A total of 10,000 events were collected. The lymphocyte percentage was determined for CD19+, CD3+, CD4+, CD8+, CD14+/CD169+, and CD11c+, and the total lymphocyte count of each subset was calculated based on the total count of lymphocytes in the spleens, mesenteric lymph nodes, and bone marrow.

For the determination of regulatory T cells, the splenocytes were re-suspended in sterile, ice-cold PBS, at a concentration of 1 × 10^7^ cells/mL. The percentage of splenic Tregs was determined using a commercial kit mouse regulatory T cell staining kit # 1. The procedure was carried out according to the manufacturer’s protocol. The cells were stained with specific mAbs, anti-mouse CD4 FITC, and anti-mouse CD25 APC (30 min in the dark, 4 °C). Then, the splenocytes were washed two times and incubated in Fixation/Permeabilization Working Solution (18 h in the dark, 4 °C). After two washes in a permeabilization buffer, the cells were stained with rat anti-mouse/rat FOXP3 PE mAb (30 min in the dark, 4 °C). Next, the splenocytes were washed two times and analyzed using a flow cytometer. The experiments also included staining with an appropriate isotype control: IgG2a PE (clone eBR2a). Fluorescence was measured by a flow cytometer (FACS Calibur; Becton Dickinson Biosciences, San Jose, CA, USA). Data acquisition and dot plot analysis were performed using CellQuest Pro software. The cells were identified as CD4+CD25+FOXP3+, CD4+CD25+FOXP3−, CD4+CD25−FOXP3+, and CD4+CD25−FOXP3− cells based on the level of CD25 and Foxp3 expression within the CD4+ lymphocyte subpopulation. The absolute count of T-regulatory cells was calculated based on the total count of splenic lymphocytes.

### 4.6. Contact Sensitivity to Oxazolone

The CS test was performed as originally described [40], with some modifications. Mice were shaved on the abdomen (2 × 2 cm area). On the next day, 100 µL of 0.5% OXA dissolved in acetone was applied to the skin of the abdomen (a sensitizing dose of the antigen). After 5 days, 50 µL of 1% OXA was applied on both sides of the auricles (an eliciting dose of the antigen). The ear edema (ear thickness) was measured by means of a spring caliper with an accuracy of 0.01 mm after 24 h from application to mice of the eliciting dose of the antigen. Three determinations from both auricles were performed for statistical evaluation. The results were presented as an antigen-specific increase in ear thickness, i.e., background (BG) values were subtracted (background values were measured in mice given only the eliciting dose of the antigen) and expressed in mm (mean/median values).

### 4.7. Analysis of the Peripheral Blood Cell Composition

The mice were put to sleep in an isoflurane atmosphere and blood was obtained from the retro-orbital plexus. The blood smears were prepared on microscopic glasses. After drying out, the smears were stained with Giemsa and May–Grünwald reagents. The smears were subsequently evaluated at 1000× magnification in a Nikon Eclipse 80i microscope (Tokyo, Japan). Up to 100 cells were counted per glass/preparation. The results were presented as a percentage of main cell types in the peripheral blood (mature neutrophils, neutrophil precursors—bands, eosinophils, lymphocytes and monocytes, and basophils). Mean values ± SE for each group were shown.

### 4.8. Immunization of Mice with OVA

On day 16, mice were immunized subcutaneously (s.c.) at the base of the tail with 5 µg of OVA emulsified in iFa, and a total suspension volume of 50 µL. After 4 days, the mice received a booster dose of OVA (50 µg) s.c. into the base of both hind legs. On day 21 after immunization the mice were bled, and sera from individual mice were isolated and frozen at −80 °C until the determination of antibody level.

### 4.9. End-Point Titrations of Anti-OVA IgG and IgG Antibodies of Each Animal Serum

A 96-well plate (Nunc MaxiSorb, ThermoScientific, Roskilde, Denmark) was coated overnight at 4 °C with 0.5 or 1 microg/mL OVA (for IgG1 or IgG, respectively) in buffered saline (PBS). The plate was then blocked for 1 h at room temperature with a blocking buffer (eBioscience™5X ELISA/ELISPOT Diluent; Thermofisher scientific, cat no 00-4202). In addition to the serially diluted (two-fold dilution series starting at 1:100) serum of each animal, the set of reference positive serum was analyzed in every experiment and its volume was used to normalize the results. A positive reference serum was prepared by pooling aliquots from 6 of the obtained control serum samples that had high antibody titers. A goat anti-mouse HRP-conjugated antibody (SothernBiotech, Birmingham, AL, USA: cat no IgG1 1071-05, IgG 1030-05) was added to the wells and incubated at room temperature for 1 h. Each serum sample was tested in duplicate. Between each step, the plate was washed four times (five times after Ab-HRP) by using the wash buffer (PBS containing 0.05% Tween 20). Then, 100 µL of TMB (Thermofisher scientific, cat no 00-4201-56) substrate solution was added to each well and incubated at room temperature for 10 min. The reaction was stopped by adding 100 μL of 2 M H_2_SO_4_ and absorbance was measured at 450/570 nm using a microplate reader. The absorbance of the reference positive serum was specified at 100% bound (Bo), and the absorbance of test samples was expressed as a percentage of Bo. End-point titration curves of each immunized mice were constructed (a five-parameter logistic (5PL, (https://www.myassays.com, accessed on April 2023) model was used). Specimens above the set cut-off (10%) were considered positive. The end-point antibody titers of sera were defined as the highest sample dilution with a B/Bo above the set cut-off value.

### 4.10. Statistics

Each experimental group consisted of 5 mice. A Brown–Forsyth’s test was used to determine the homogeneity of variance between groups. When the variance was homogenous, an analysis of variance (one-way ANOVA) was applied, followed by post-hoc comparisons with Bonferroni or Tukey’s tests to evaluate the significance of the difference between groups. Nonparametric data were evaluated with Kruskal–Wallis’s analysis of variance. The results are presented as mean values ± standard error (SE) or mean/median, Q1–Q3 range and min–max values. Significance was determined at *p* < 0.05. The data were analyzed statistically using STATISTICA for Windows software (version 13.3).

## Figures and Tables

**Table 1:** **The composition of peripheral blood cell types upon determination of contact sensitivity to oxazolone.** The mice were bled and the blood smears were stained with May-Grünwald as well as Giemsa reagents, followed by a review by a histologist. The following cell types were analyzed and were presented as a percentage of content (mean values from 5 determinations; 2 smears/mouse ± SE): immature neutrophils (bands; B), mature neutrophils (segments; S), eosinophils (E), lymphocytes (L), monocytes (M), basophils (B); * statistically significant in comparison to control mice’s drinking water and not sensitized with OXA (water/BG group).

Experimental Group	% of Cell Types in the Circulating Blood					
	B	S	E	L	M	B
water/BG	1.5 ± 0.0	32.1 ± 1.3	1.5 ± 0.2	62.2 ± 0.4	2.0 ± 0.4	1.0 ± 0.2
water/OXA	3.4 ± 0.9	48.5 ± 2.8 *	1.5 ± 0.2	41.2 ± 2.6 *	3.8 ± 1.1	1.5 ± 0.2
yolkin/OXA	2.7 ± 0.6	52.9 ± 0.8 *	1.0 ± 0.2	39.3 ± 1.2 *	1.9 ± 0.5	1.4 ± 0.2

**Table 2:** **Phenotypic changes in lymphoid organs of mice treated with yolkin from the 28th day of life.** Mice with a mean age of 28 days were administered yolkin in their drinking tap water, at a concentration of 5 µg/mL, for 15 consecutive days. In the morning, after discontinuing the yolkin application (15th day of test), lymphoid organs were taken to determine any phenotypic changes. The results are presented as a percentage of selected lymphocyte subpopulations and macrophages (mean values from 5 determinations ± SE); * statistically significant in comparison to control, water-drinking mice.

Splenocytes						
	CD8+	CD4+	CD3+	CD19+	CD14+CD169+	CD25+Foxp3+
Control	1.69 ± 0.22	9.56 ± 0.94	18.32 ± 3.11	69.29 ± 6.03	2.53 ± 0.12	10.48 ± 0.36
Yolkin	1.42 ± 0.13	11.23 ± 0.45	15.31 ± 0.97	75.94 ± 0.88	1.91 ± 0.34	8.48 ± 0.20 *
Mesenteric lymph node cells						
	
	CD8+	CD4+	CD3+	CD19+		
Control	5.39 ± 0.35	42.10 ± 1.35	48.61 ± 1.37	49.19 ± 1.28		
Yolkin	8.59 ± 0.52 *	50.22 ± 2.01 *	55.97 ± 1.43 *	42.62 ± 1.43 *		
Bone marrow cells						
		
	CD19+		CD11c+			
	Gated for lymphocytes only	Gated for all cells				
Control	82.62 ± 0.82	36.83 ± 1.76	2.83 ± 0.22			
Yolkin	84.95 ± 1.93	40.70 ± 2.32	2.25 ± 0.35			

**Table 3:** **Phenotypic changes in lymphoid organs of juvenile mice treated with yolkin from the 37th day of life.** Mice with a mean age of 37 days were administered yolkin in their drinking tap water, at a concentration of 5 µg/mL, for 15 consecutive days. In the morning, after discontinuing the yolkin application (15th day of test), lymphoid organs were taken to determine any phenotypic changes. The results are presented as a percentage of selected lymphocyte subpopulations and macrophages (mean values from 5 determinations ± SE); * statistically significant in comparison to control, water-drinking mice.

Splenocytes						
	CD8+	CD4+	CD3+	CD19+	CD14+CD169+	CD25+Foxp3+
Control	2.81 ± 0.22	17.55 ± 1.52	22.62 ± 2.49	68.99 ± 2.33	3.30 ± 0.35	9.85 ± 0.40
Yolkin	2.99 ± 0.14	16.50 ± 1.67	20.44 ± 2.31	66.43 ± 2.11	3.58 ± 0.13	8.12 ± 0.21 *
Mesenteric lymph node cells						
	
	CD8+	CD4+	CD3+	CD19+		
Control	7.91 ± 0.66	45.81 ± 2.67	49.86 ± 3.22	47.78 ± 3.24		
Yolkin	4.69 ± 0.17 *	42.47 ± 1.08	44.40 ± 2.06	52.97 ± 1.73		
Bone marrow cells						
		
	CD19+		CD11c+			
	Gated for lymphocytes only	Gated for all cells				
Control	73.09 ± 1.65	30.91 ± 0.89	4.62 ± 0.65			
Yolkin	77.63 ± 1.40	32.55 ± 1.51	5.45 ± 0.24			

## Data Availability

Data are available from authors on request.
